# Systems-Level Mapping of Cancer Testis Antigen 1b/a to Sarcoma Pathways Identifies Activated Ran Binding-2 E3 SUMO-Protein Ligase and Transducin-Like Enhancer Protein 1

**DOI:** 10.3389/fgene.2022.834445

**Published:** 2022-05-18

**Authors:** Nikolaos A. Papanikolaou, Prodromos Hytiroglou, Pavlina Pantelidou, Athanasios G. Papavassiliou, Lloyd L. Old

**Affiliations:** ^1^ Laboratory of Biological Chemistry, Department of Medicine, Section of Biological Sciences and Preventive Medicine, School of Medicine, Aristotle University of Thessaloniki, Macedonia, Greece; ^2^ Department of Medicine, Laboratory of Pathology, School of Medicine, Aristotle University of Thessaloniki, Macedonia, Greece; ^3^ Department of Biological Chemistry, Medical School, National and Kapodistrian University of Athens, Athens, Greece; ^4^ Ludwig Institute for Cancer Research, Memorial Sloan Kettering Institute for Cancer Research, New York City, NY, United States

**Keywords:** CtaG, GSEA, network, sarcoma, RanBP2, TLE1, transcription

## Abstract

Here we describe the identification of genes and their encoded proteins that are expressed in advanced grade tumors by reconstruction of a sarcoma cancer testis gene 1b/a (*catg1b/a*) network. CTAG1B/A is an ortholog of the yeast/Drosophila transcription factor Pcc1p, and a member of the KEOPS transcription complex. It has been implicated in telomere maintenance and transcriptional regulation through association with chromatin remodeling factors and is only expressed during adult testis germ cell differentiation. C*tag1b/a* is re-activated in synovial sarcomas and myxoid liposarcomas but not in differentiated liposarcomas. We mapped CTAG1B/A protein to sarcoma transcription pathways with gene set expression analysis (GSEA) and using independent samples, we immunohistochemically identified expression of at least two network neighbors, RANBP2, and TLE1, thus validating our approach. This work demonstrates that mapping unknown genes to functional pathways by network re-construction is a powerful tool that can be used to identify candidate oncoproteins.

## Introduction

The cancer testis genes (*ctags*) are a heterogeneous group of genes, that is, exclusively expressed in gametogenic germ cells (Ait-Tahar et al., 2009), and trophoblasts (Chang and Nevins, 2006), but is aberrantly re-expressed in different tumors (Ait-Tahar et al., 2009; Cho et al., 2006; Cooke and Nanjappa, 2015). The absence of expression in normal adult tissues makes these genes and their protein products unique as therapy targets and as potential diagnostic tools or markers (DeRisi and Iyer, 1999; DeRisi et al., 1997; Epping et al., 2005). With the exception of the SCP-1 (Fordham et al., 2020) and OY-TES-1 CT proteins (Gioutlakis et al., 2017), virtually nothing is known about their biological functions either in gametogenesis, trophoblast biology or tumor development. Identifying the biological functions of *ctag* gene products by mapping them to active pathways is therefore critical for defining their role in normal development and in cancer growth. In addition, analysis of their networks, will facilitate the identification of novel gene products with hitherto unknown functions (Gnjatic et al., 2006; Gjerstorff et al., 2007; Gjerstorff et al., 2007). A serious obstacle to this endeavor, however, is the random patterns of expression in tumors and the relative lack of human germ cell development models (Hecker et al., 2008; Grigoriadis et al., 2009).

*Ctag1b/a* was the first cancer testis antigen gene to be discovered in cancer patients using an *in vivo* antibody response and successfully targeted by a vaccine (Hei et al., 2019). It is localized on the Xq28 region of the X chromosome encoding a 180 long amino acid protein. *Ctag1b/a* is found in approximately one third of all melanoma, lung, and esophageal tumors, from which it was first isolated, as well as in liver, gastric, prostate, ovarian and bladder cancers (Henderson et al., 2005). It occurs in 80% of synovial sarcomas, a rare but highly aggressive tumor type (Hua et al., 2014), and also in myxoid liposarcomas (Iura et al., 2015; Irie et al., 2018), a type of aggressive mesenchymal tumor but not in well differentiated liposarcomas (Jiang et al., 2017). Single and double immunohistochemical studies in human germ cells have revealed that CTAG1B/A protein is associated with highly proliferating germ cells (Jungbluth et al., 2001). Its expression during male germ development is limited to a narrow window spanning mitosis and meiosis (I) events at the types A and B spermatogonium stages (Kakimoto et al., 2019). In melanoma cells the CTAG1B/A protein is found in complexes with MAGEC1, another cancer testis antigen protein and a putative transcription factor, which is consistent with the narrow window of expression of both proteins in differentiating germ cells. The CTAG1B/A protein is a homolog of the yeast Pcc1p transcription factor (Kisseleva-Romanova et al., 2006)] and a member of EKC complexes, which control the yeast cell cycle, raising the possibility that CTAG1B/A is a germ cell-specific transcription factor. The above data suggest that the two proteins may have common functions mediated by a CTAG1B/A/MAGEC1/KEOPS complex (Kalejs and Erenpreisa, 2005).

We reasoned that by extracting gene and transcriptional modules that are co-expressed or co-regulated with *ctag1b/a*, we could map it to tumor pathways and use its network properties to identify network neighborhood genes that are also expressed in sarcoma samples (Klapa et al., 2013; Kobayashi and Surani, 2018). Functional modules of co-expressed genes reflect coherent changes in biological properties beyond individual genes and characterize the behavior of cells at higher levels of organization (Liggins et al., 2010). Using transcriptomics samples, we have extracted *ctag1b/a* co-expression modules from different tumor types in which *ctag1b/a* is re-expressed, and reconstructed a sarcoma *ctag1b/a* network. Next, we identified several network neighbors that are candidates involved in sarcoma biology. Using different sarcoma samples, we immunohistochemically confirmed expression of some of the *ctag1b/a* network neighbors. *Ctag1b/a* is enriched in transcription factor pathways, suggesting that it is likely a germ cell-specific transcription factor, that is, re-expressed, albeit randomly, in adult sarcomas and in various other tumors. Thus, by using reverse network engineering, we demonstrate that it is possible to discover and map co-expressed genes in sarcoma biology pathways.

## Materials and Methods

### Microarray Data Collection and Analysis of *Ctag1b/a* Differential Expression

Gene expression data were downloaded as text (txt.) files from the Gene Expression Omnibus (GEO) at NCBI. 32 microarrays deposited at NCBI’s GEO or EBI’s Array Express databases were retrieved and analyzed for *ctag1b/a* differential expression between normal vs. primary or normal vs. metastatic tissue, and isolated glioblastoma stem cells vs. normal adult human tissues (Supplementary File S1).

### Mining Interactions and Interrelationships

First and second level interactors of the CTAG1B/A protein were extracted from the PICKLE database (Klapa et al., 2013; Gioutlakis et al., 2017) which contains comprehensive data from all known interaction databases and integrates knowledge with genetic data. Data were filtered for confirmed physical/functional interactions and, in order to minimize false positives, only those with an E value > 0.7 were retained.

### Extraction of Co-regulated *Ctag1b/a* Gene Modules From Sarcoma Samples

*Ctag1b/a* co-expression gene modules were extracted from synovial and myxoid (grade 3) sarcomas as well as various benign fibrosarcomas (grade1-2) using the three step strategy shown in Figure 2A (Supplementary Appendix SB for the computational methods). Our working hypothesis was that genes that are co-expressed in sarcomas with *ctag1b/a* are also likely to be co-regulated if they are in the network neighborhood of *ctag1b/a*. We implemented our strategy as follows: First, we established whether *ctag1b/a* is differentially expressed between two different classes of samples, such as, for example, between normal vs. metastatic tissue, benign vs. primary tumor, etc, (Supplementary File S1). Next, we subjected the samples to pair-wise analysis using either Student’s t test (null hypothesis: The medians/means of expression between two classes of samples are not significantly different at *p* < 0.01) or Mann-Whitney’s non-parametric statistic for rank distribution in order to find if the expression levels of *ctag1b/a* between two sample classes differed significantly between classes. We have found that *ctag1b/a* was induced at significant levels in eight out of 32 downloaded microarrays (*p* < 0.01, Supplementary File S1, datasets where *ctag1b/a* is induced significantly are in red). Microarray data with *p* > 0.01 were rejected. The choice of the two tests depended on the number of replicates in each sample class. Only three sets of data passed the strict cutoff point *p* = < 0.01, microarrays GDS1209a and b and GDS2736. A second reason for rejecting the other data was the random distribution of *ctg1b/a* re-expression in both advanced (metastatic) and non-metastatic tumor samples. We then derived gene modules that are co-expressed either positively or negatively with *ctag1b/a* using gene set enrichment analysis (GSEA) as described below.

### Gene Set Enrichment Analysis for Gene Sets Showing Co-Regulation With Ctag1b/a

We extracted ctag1b/a gene modules in two steps, as described in the previous section. Next, we used gene set enrichment analysis (GSEA, Supplementary Appendix SB) to obtain gene sets enriched either for CTAG1B/A_POS or negative expression, (CTAG1B/A_NEG) (Parameters and results in Supplementary File S2).

### *Ctag1b/a* Synovial Network Reconstruction

The retrieved binary interaction data from the PICKLE database were collected in an Excel file and used a txt file in Cytoscape. Only data with an E value equal or greater than 0.7 were considered. Using as queries the sarcoma genes in the GSEA-extracted modules, the genes found in the indicated gene collections (Papanikolaou gene sets compiled by the Papanikolaou lab, Figure 2), mitosis-meiosis gene sets, and the genes in the Coxpred database that are known to be co-regulated with *ctag1b/a* as well as their first and second-level physical/genetic interactors, we reconstructed a *ctag1b/a* synovial network with Cytoscape and analyzed its neighborhood network properties using the MCODE algorithm for clustering highly interconnected genes and for finding multiprotein complexes (modules) and also cytoHubba for identifying hub genes and sub-networks in the neighborhood of *ctag1b/a*.

### Gene Ontology Analysis

We subjected the top 20 genes from each of the CTAG1B/A_POS and CTAG1B/A_NEG modules to systems-level gene ontology (GO) analysis with the GATHER algorithm (Chang and Nevins, 2006). Enrichment for functional GO pathways was performed based on statistical significance using a GATHER algorithm-based Bayesian approach (Supplementary Files S4, S5, respectively).

### Immunohistochemistry

Sections of formalin-fixed, paraffin-embedded tissues derived from synovial sarcomas were obtained from the archives of the Pathology Laboratory, Department of Medicine, Aristotle University. Ganlioneuroma IHCs were a gift from Dr. Achim Jungbluth, Laboratory of Pathology, Ludwig Institute for Cancer Research in New York, United States. In all cases, slides were reviewed and representative blocks were chosen for immunohistochemical analysis. The following primary monoclonal (MAb) or polyclonal (PAb) antibodies were used at concentrations ranging from 1:200 to 1:1,000 (primary antibody): CTAG1B/ (sc-53869), CT7-33 mouse MAb against MAGEC1 (sc-53868), Ranbp2 (ABCAM, PAB 2938TRS), TLE1 (ABCAM, PAb, 15587syn), MDM2 (Santa-Cruz Biotechnology, D-7, sc-13161). Tissue sections were deparaffinized in xylene and graded alcohols. The stains were performed in an automated staining system (Bond-max, Vision Biosystems, Germany). For antigen retrieval the slides were heated at 99°C in a 10 mM sodium citrate solution (pH 6.0) for 10 min using a vegetable steamer (Oster 5712 food steamer, Maitland, Florida, United States). Labeling was visualized using 3, 3′-diaminobenzidine (DAB, Vector Laboratories, Burlingame, California, United States) or NovaRED (Vector Laboratories). Alternatively, the Thermo Fisher Scientific MultiVision Polymer Detection System (Thermo Fisher Scientific, Lab Vision Corporation, Fremont, California, United States) was used for detection, and development was achieved using 3, 3′-diaminobenzidine (Vector Laboratories) followed by application of NovaRED (Vector Laboratories). Slides were counterstained with Mayer’s haematoxylin. For negative controls non-immune serum of the same species was applied as primary antibody.

## Results

### Re-Activation of Ctag1b/a Expression in Different Tumors

**FIGURE 1:**
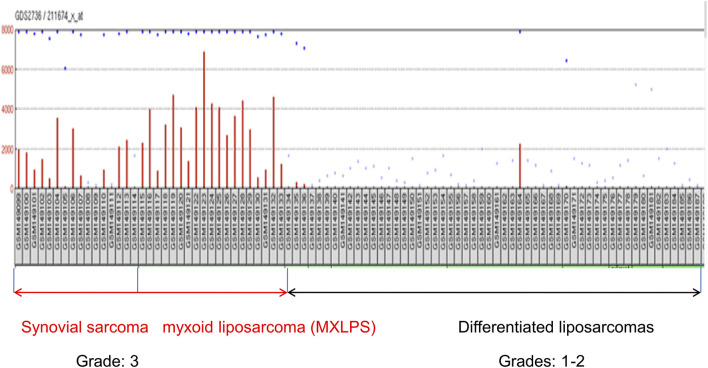
Expression of *ctag1b/a* mRNA in different sarcoma samples retrieved from the GDS2736 archive. Notice that with the exception of two samples, various differentiated sarcomas of grades 1–2 uniformly lack expression of *ctag1b/a*. In contrast, *ctg1b/a* is re-expressed in most (grade 3) synovial and myxoid liposarcomas samples as well as dedifferenatiated liposarcomas. Expression unitson the Y axis are arbitrary transcriptomics units. Retrieved samples are designated by GSM, followed by a number.

**FIGURE 2:**
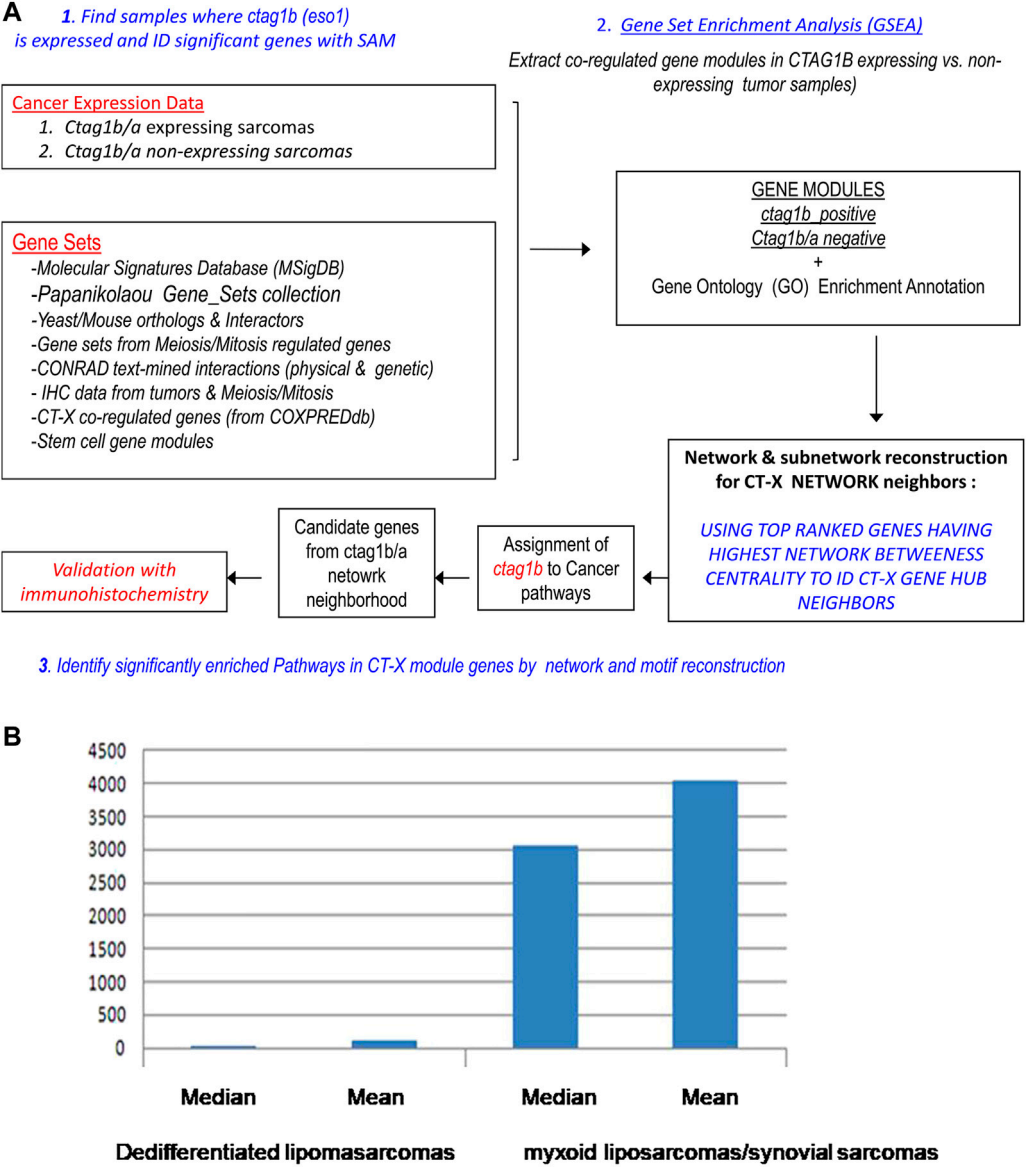
**(A)** Strategy for the extraction and characterization of synovial *ctag1b/a* co-expression modules, reconstruction of a synovial *ctag1b/a* network and identification of its neighbor genes in the network. **(B)** Median and Mean values of *ctag1b/a* expression in differentiated lipοsarcomas or myxoid liposarcomas/synovial sarcomas. Pairwise comparison was with the T statistic (*p* value was equal to 3.3 × 10^–7^). The null hypothesis is that there is no difference in *ctag1b/a* expression between the two groups of tumors. Expression units on Y axis are arbitrary transcriptomics units.

In order to map *ctag1b*/a to cancer pathways and to discover co-expressed genes in its network neighborhood, we exploited its re-activation in synovial and myxoid liposarcomas but not in dedifferentiated liposarcomas or myofibrosarcomas and leiomysosarcomas (Figure 1) and mined its physical and genetic interactions. We then re-constructed a *ctag1b/a* sarcoma network and used its properties to identify genes in the neighborhood of *ctag1b/a*. It has been reported that expression of *ctag1b/a* (aliases include: *ny-eso-1*, *ct6.1*, *ctag2*, *lage-2*, *lage-2a* and *lage2b*) is re-activated, albeit randomly, in several different tumor types but without the distinct expression profile seen in sarcomas (Supplementary File S1, Figure 1). We therefore focused on sarcoma samples and using the three-step strategy shown in Figure 2, we extracted *ctag1b/a* co-expression modules (step 2 in Figure 2A), as described in Section 2. We then subjected the gene lists to network analysis and identified *ctag1b/a* network neighbors and key hubs linked with shortest paths (with the cytoHubba algorithm in Cytoscape) in the *ctag1/a* neighborhood. Finally, using immunohistochemical staining of separate, independent samples, we examined the protein expression status in independent sarcoma or control ganglioneuroma samples and confirmed their expression. Two of these genes, *tle1* and *ranbp2*, have previously been implicated in sarcoma biology, thus validating our approach. Our findings have potential applications for the identification of novel synovial sarcoma sub-networks and genes with hitherto unknown functions.

The *ctag1b/a* query in the Gene Expression Omnibus (GEO) database returned 4033 *ctag1b/a* expression profiles for different tumor types. Of these, only thirty two (Scanlan et al., 2002b) merited further examination (Supplementary File S1). Ten out of thirty two (31%) microarray samples exhibit significant (*p* < 0.05) differences in *ctag1b/a* expression between samples (Supplementary File S1). Microarray data with *p* ≥ 0.01 were not considered further. In contrast, in two studies (GDS2736 and GDS1209) almost all samples of stage 3 synovial sarcomas and myxoid liposarcomas uniformly express *ctag1b/a* whereas various differentiated liposarcomas do not show re-activation (Figure 1 and Table 1). With the exception of sarcoma, melanoma and CD133 positive glioblastoma samples, *ctag1b/a*, is randomly re-activated in most samples examined and does not follow any pattern, that is, characteristic of stage or tumor type. Thus, in three melanoma studies (GDS 1375, GDS 1965, and GDS1078) *ctag1b/a* is re-activated in 33% of samples. Of the fourteen breast cancer samples only one group (GSE3156) exhibits re-activation. All others display a non-uniform, dispersed re-expression pattern (Cooke and Nanjappa, 2015). On the other hand, 33% of CD133 positive glioblastoma samples exhibit re-activation whereas CD133 negative ones do not re-express it at all.

**TABLE 1:** Different tumor types used in extracting *ctag1b/a* modules from trascriptomics data. Shown in red are samples of advanced sarcoma types as well as melanomas that express *ctag1b/a*. Samples were grouped by tumor type and ctag1b/a expression status.

Sample grouping of tumors by *ctag1b/a* expression status		
Tumor type	Number of samples	ctag1b/a expression status
Primary/normal melanoma	3	No
nNSCLC tissue (lung cancer cell lines/non-metastatic	3	No
Lipomas	16	No
Fibromyosarcoma	8	No
NSCLC	3	Yes
Myxoid liposarcoma	15	Yes
Synovial sarcoma	10	Yes
Metastatic melanoma	5	Yes
Total	63	—

We focused on sarcoma samples because they exhibited a distinct *ctag1b/a* expression pattern. We subjected the different groups of samples to pair-wise analysis using either the Mann-Whitney non-parametric statistic for rank distribution of *ctag1b/a* expression levels, or Student’s paired t-test (Figure 2B). The choice of the two tests depended on the number of replicates in each sample class. *Ctag1b/a* was found to be significantly overexpressed (*p* < 10^–7^) in myxoid liposarcomas and synovial sarcomas but not in lipomas or leiomyosarcomas, a significant finding.

### Identification of Ctag1b/a Synovial Re-Expression Gene Module Signatures

Using GSEA we extracted two phenotypic classes from a compendium of ten sarcoma types in two studies (GDS1209), representing gene modules whose co-expression pattern either correlates (CTAG1B/A-POS) or anti-correlates (CTAG1B/A_NEG) with *ctag1b/a* re-expression (data in Supplementary Files S2, S3). The GSEA-generated phenotypic classes (No expression vs. expression, Supplementary Figures S1A,B) were separated according to their GSEA-computed enrichment score (ES) and plotted against their *p* value. *Ctag1b/a* was used as an index gene (details in Computational Methods section; Supplementary Files S2, S3 contain all genes identified above a cutoff threshold normalized enrichment score (NES) (NES = enrichment score normalized to mean enrichment of random samples of the same size) as having either a positive or negative correlation with *ctag1b/a*, the index gene in GSEA. The extracted gene set-containing phenotypic classes were assigned a positive or negative enrichment score (ES, Supplementary Figure S1A, upper left panel, A) depending on their rank position in the Kolomogorov-Smirnov list (Supplementary Figure S1A, lower middle panel).

Genes that are enriched in the CTAG1B/A-POS phenotype are up-regulated (positive ES), whereas those in the CTAG1B/A_NEG phenotype are down-regulated, hence the negative ES. The gene sets within each phenotype represent actual biological pathways involved in processes enriched with respect to *ctag1b/a* re-activation and are assigned by GSEA a NES and a *p* value which determine their position in the ranked list (Supplementary Files S2, S3). The lower the NES value the more significant the gene set. For example, the top gene set enriched in this phenotype is the GPCRD_rhodopsin_like set with 169 gene members and a NES of −3.8 (see *p*-value plots sheet in Supplementary File S2). *Ctag1b/a* was assigned a score equal to zero and was used as an index gene in GSEA, followed by computation of the KS sum for all genes in the array. The smaller the KS sum for a particular gene the closer that gene is to *ctag1b/a* in terms of expression profile enrichment. On the other hand, the larger the KS score the more distant in terms of expression profile is the gene. Thus, the gene closest to *ctag1b/a* in this list is LOC349160 (Supplementary File S3), an RNA gene, and belongs to an lncRNA class in the human genome followed by the rest in the list going downwards. Of the 1,245 gene sets in the MSig database, 1,171 are positively upregulated with *ctag1b/,* whereas only 74 of 1,245 are downregulated in the negative phenotype (Supplementary File S2), suggesting that *ctag1b/a* re-activation is linked to many activated sarcoma pathways. Notable among the twenty five top genes found to be co-expressed with *ctag1b/a* in sarcomas are protocadherin β3, tumor necrosis factor alpha-induced protein 8-like 3, demethylase jumonji domain containing 2C, cyclin-dependent kinase 6, and glycine dehydrogenase (decarboxylating) (Supplementary File S3).

Gene ontology enrichment (GO) analysis showed that the CTAG1B/A*-*POS gene module is almost exclusively enriched in genes for transcription factor activity (Supplementary Figure S1A, lower right panel). In contrast, the CTAG1B/A*_*NEG module (Supplementary Figure S1A, lower right panel) is enriched in structural and macromolecular synthesis genes such as ribosomal genes. Several genes in the CTAG1B/A*-*POS module, such as *mdm2*, and *cdk6*, have established oncogenic roles, whereas others remain uncharacterized. Examples include the zinc finger gene 588 (*znf588*), protocadherin β3, laminin α1 and cyclin b3 (Supplementary File S3, sheet 1: 50 co-expressed genes/Heat Map).

Although the genes in each CTAG1B/A*-*POS module in the microarray datasets were different, their enrichment with KEGG pathways, GO functions and transcription factor binding sites (Supplementary Figure S1A, upper right panel) suggested that they belonged to gene groups involved in morphogenesis (GO:0009653), cell motility, organogenesis, cell migration etc, as indicated by their decreasing ln (Bayes factor) [Supplementary File S4 and 5 under ln (Bayes factor)]. In contrast, the CTAG1B/A*_*NEG module consistently contained ribosomal genes in all ten microarrays (Supplementary File S4). GO annotation enrichment analysis of these genes returned protein biosynthesis (GO: 0006412), macromolecular biosynthesis and other enriched annotations as top functional categories.

### Re-Construction of a Ctag1b/a Synovial Network

**FIGURE 3:**
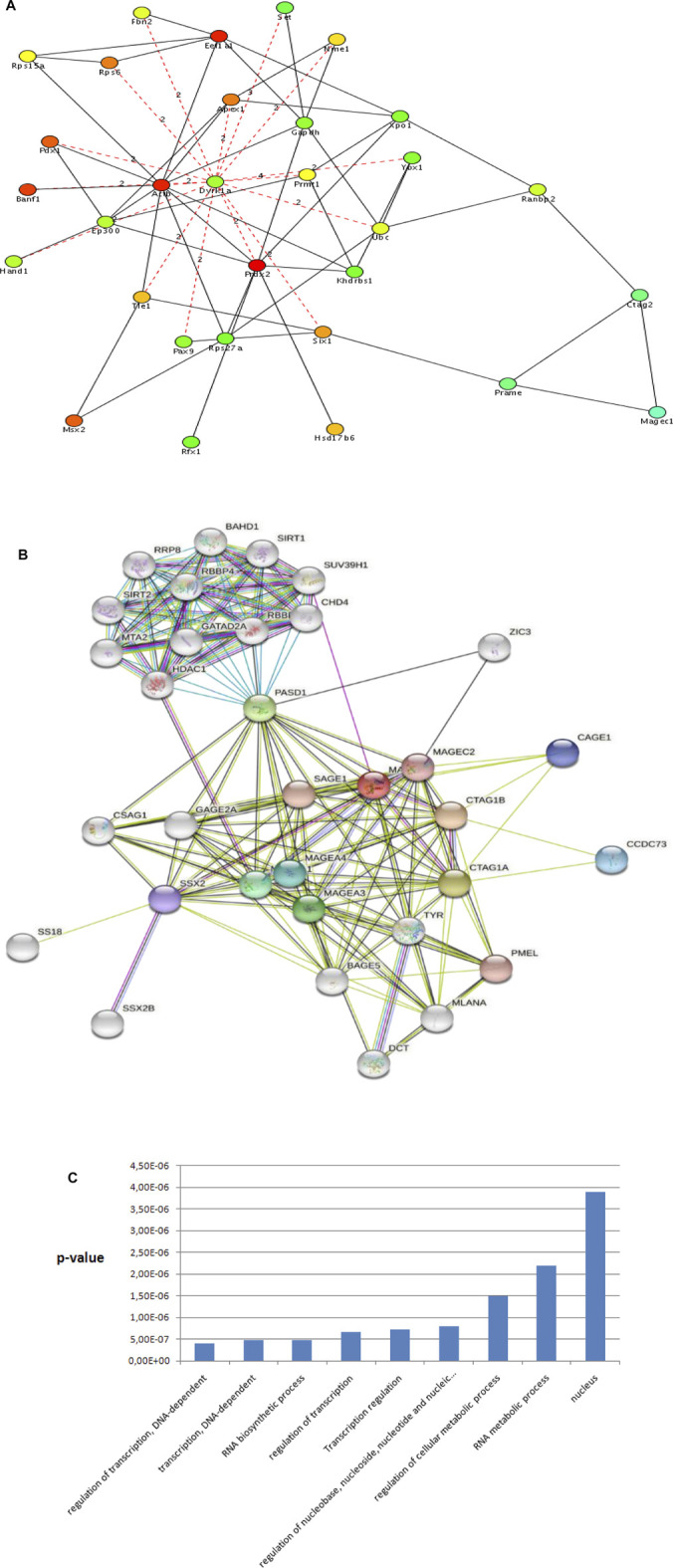
**(A)** *Ctag1b/a* STRING network. *ctag1b, ctag1a* are aliases of *ctag1b/a*. The network neighborhood was reconstructed from first and second level interactors of *ctag1b/a*, *pasd1* and ZIC3 are transcription factors. *Pasd1* links the *ctag1b/a* group (lower group containing the *mages, cage1* and others) with chromatin accessory proteins (upper group containing *hdac1, chd4* and others). Network was generated with Cytoscape. **(B)** The ctag1b/a network neighborhood. Shortest paths, shown in broken lines, between *ctag1b/a, magec1, prame* and top important genes in the network (Table 2) were calculated with the Dijkstra algorithm within Cytoscape. Top ranked nodes/bottlenecks are shown in different colors with each rank in a different color with highest ranked nodes in red, followed by pink and green for lower ranked nodes. Dashed red lines are calculated shortest paths between nodes in the c*tag1b/a* network neighborhood in sarcoma samples. Solid lines: Functional or physical edges (links) between nodes. Broken lines: Shortest paths (computed with the Dijkstra algorithm within Cytoscape) from the network in Panel 3A. *Ctag2* is an alias of *ctag1b/a* (also *ctag1b* or *ctag1a*). Expression of *tle1*and *ranbp2* was tested in independent archived synovial sarcomas (Laboratory of Pathology, Aristotle University School of Medicine, Macedonia, Greece) and ganglioneuromas (Ludwig Institute for Cancer Research, New York, NY United States). The networks were generated with Cytoscape. **(C)** Top GO categories for genes in *ctag1a/magec1* network neighborhood (Panel 3A and Table 2). Note: Regulation of transcription and Transcription regulation GO categories contain complementary though not identical genes. *p* values were calculated with the hypergeometric method using random sets of genes from the GO database as reference sets shown in Panel 3B.

Using the genes and their first and second-level physical and genetic interactors (Figure 2, step 2), we constructed a *ctag1b/a*-centered sarcoma network with Cytoscape. The sarcoma network consists of 1,140 nodes (genes), and 7,972 edges (interactions/interrelationships). It has a clustering coefficient equal to 0.163 and an average path length equal to 3.443 (Figure 3A shows part of the *ctag1b/a* network) values that are typical for biological networks. Notable among members of this network is *pasd1* which encodes a transcription factor linking a group of genes encoding a cluster of chromatin proteins such as *hdac1*, *chd4*, *mta1* (Figure 3A, upper group). *Pasd1* and *zic3* link the chromatin gene cluster with the *ctag1b/a* neighborhood and *with magec2*.

**FIGURE 4:**
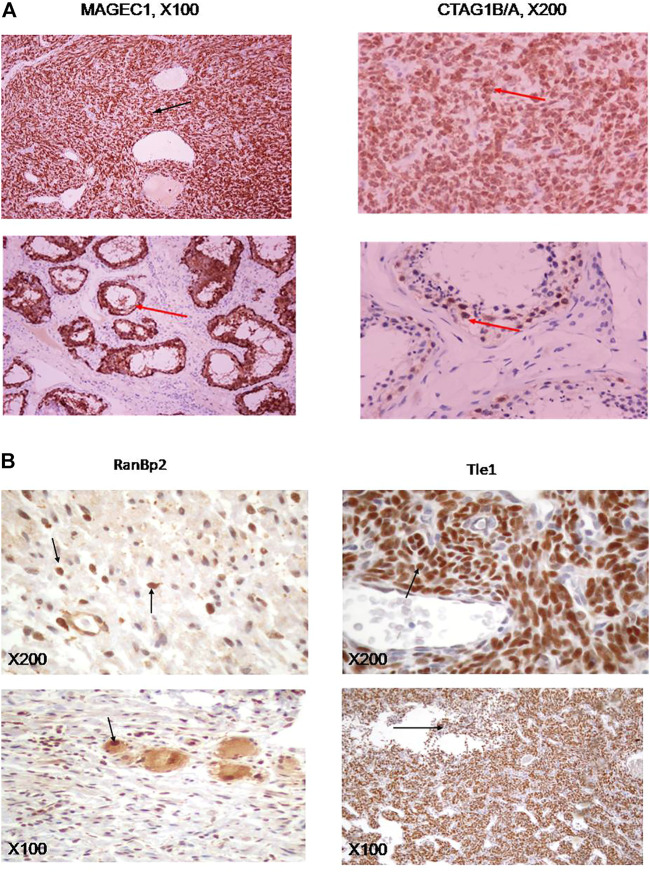
**(A)** Immunohistochemical stains of synovial sarcoma specimens for MAGEC1 (left panel), or CTAG1B/A (right panel), as well as appropriate controls (testicular tissue, lower panels). **(B)** Immunohistichemical stains of ganglioneuroma samples for RANBP2 (left panels) and synovial sarcomas for TLE1 (right panels). Antibodies used are described in Materials and Methods. Magnification was at 100× (upper panels) or 40× (lower panels) on a NIKON Diaphot 200/300 inverted microscope.

Several features are apparent in the sub-network neighborhood in Figure 3B: First, the network neighborhood suggests that *ctag1b/a* (aliased as *ctag2* in the sub-network), *prame* and *magec1*, form a clique. Also, *prame* is linked to *six1* and *ctag2* to *ranbp2*. Top bottlenecks were computed using Dijkstra’s algorithm in Cytoscape and the top physical complexes were extracted with the MCODE algorithm. Lastly, we calculated the shortest paths between the top 20 hubs (Table 2 and Figure 3B), including *ctag1b/a* in the *ctag1b/a* network neighborhood (Figure 4B). The rationale behind this approach was based on the fact that top nodes (hubs) that are also bottlenecks organize biological networks into broader functional groups and contribute to network stability (robustness). On the other hand, shortest paths (Figure 3B, broken lines) among top network bottlenecks indicate how close nodes (genes/proteins) are in a network and therefore can help map unknown genes to pathways or suggest biological roles as well as indicating that signal transduction is efficient. Notably, *prame, six1* (a known oncogene), *magec1* and *ranbp2* (a large GTP binding protein) are close neighbors of the *ctag1b/a* sub-network (Figure 3B). Interestingly, though shortest paths to *ctag1b/a* go through *dyrk1a*, suggesting a central role for this gene in this sub-network (Figure 3B), we were not able to procure antibodies to test its presence. GO analysis shows that the *ctag1b/a* neighborhood is enriched in genes involved exclusively in transcription (Figure 3C). Lastly, the *ssx2* gene, that is, found in the *ctag1b/a* subnetwork (Figure 3A), is involved in 80% of all synovial sarcomas as fusion protein products SSXT-SSX1 or SSXT-SSX2, derived from translocation t (X; 18) (p11.2; q11.2).

**TABLE 2:** Important synovial sarcoma nodes identified with Cystoscape. Nodes found to be hubs (important nodes with numerous edges) and close expression neighbors of *ctag1b/a* with the gene set enrichment (GSEA) module analysis method are shown in red. Prdx2 is highlighted yellow as the top-ranked node.

Name	Score	Rank
Prdx2	46.490.800.000	1
Actb	28.369.400.000	2
Eef1a1	10.148.500.000	3
Banf1	9.006.900.000	4
Msx2	7.618.400.000	5
Pdx1	7.540.200.000	6
Apex1	7.087.200.000	7
Rps6	6.248.100.000	8
Six1	5.960.900.000	9
Hsd17b6	5.881.600.000	10
Tle1	5.716.000.000	11
Nme1	5.370.300.000	12
Prmt1	5.295.800.000	13
Rps15a	5.137.900.000	14
Ubc	5.057.900.000	15
Fbn2	4.915.400.000	16
Ranbp2	4.616.200.000	17
Hand1	4.609.600.000	18
Ep300	4.241.300.000	19
Dyrk1a	4.204.200.000	20
Gapdh	3.295.900.000	21
Pax9	3.096.700.000	22
Xpo1	3.056.900.000	23
Ybx1	2.976.900.000	24
Rfx1	2.928.000.000	25
Khdrbs1	2.921.900.000	26
Rps27a	2.640.800.000	27
Set	2.640.300.000	28
Hdm2	2.560.800.000	29
Anp32a	2.498.400.000	30

### Validation of Expression of Ranbp2 and tle1 in Sarcoma Samples by Immunohistochemistry

We confirmed expression of some of the *ctag1b/a* network neighbors in advanced grade sarcomas (Figure 3B and Table 2) by using independent samples from the Department of Pathology at Aristotle University School of Medicine. Immunohistochemical staining for MAGEC1, a protein, that is, found in intracellular complexes with CTAG1B/A, showed extensive (80%) positivity in synovial sarcoma cells in one of seven cases evaluated (Figure 4A), whereas the remaining six cases were negative. On the other hand, staining for CTAG1B/A showed variable positivity of synovial sarcoma cells in six of seven cases. The CTAG1B/A positivity was extensive in three cases, involving 90–100% of the neoplastic cells (Figure 4A), and focal in the other three cases, involving 7–25% of the cells (data not shown). Interestingly, staining for MDM2 control revealed extensive positivity (data not shown).

RANBP2 and TLE1 exhibited extensive positivity in several different, independent ganglioneuromas (samples DSCN1432-1,436, Figure 4B), albeit with a different pattern. In the case of RANBP2, there are small yet distinct individual cells scattered throughout the field (Figure 4B, left upper and lower panels) whereas in the case of TLE1, the entire field of vision is covered with positive cells (results not shown), similar to synovial sarcomas (Figure 4B, upper right and lower panels). The lack of resources prevented us from testing expression of some of the other highly promising candidates such as PASD1 and DYRK1A.

## Discussion

**FIGURE 5:**
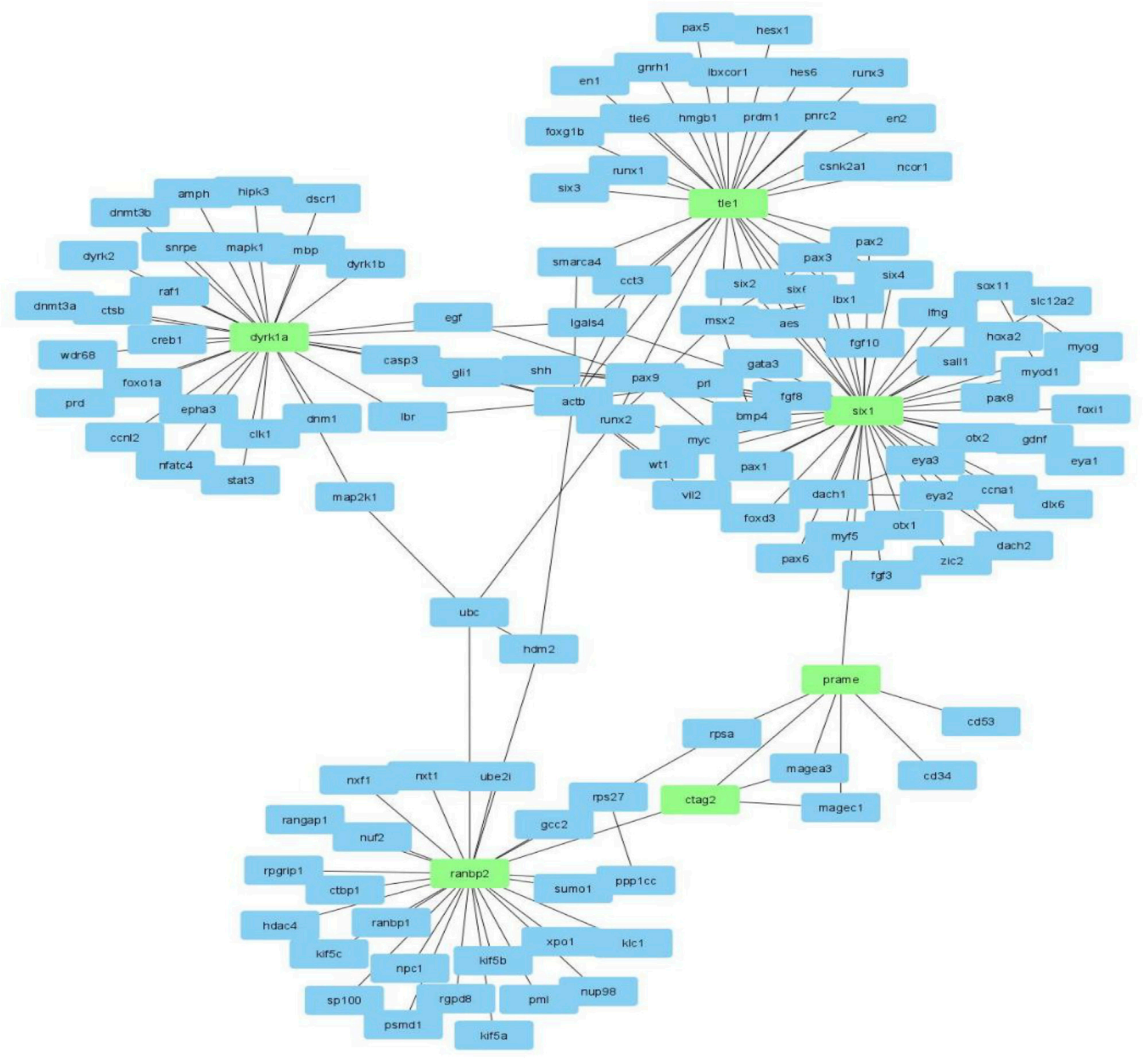
The *ctag1b/a* (*ctag2*) network neighborhood rearranged with Cytoscape in order to reveal the links and neighborhoods between *ctag1b/a*, *ranbp2*, *prame*, *six1*, *tle1* and *dyrk1a*.

Mesenchymal tumors that arise from soft tissues are rare and belong to a varieity of subtypes, hindering our understanding of the underlying molecular pathology. In this work we have identified *ranbp2* and *tle1* as co-expressed network neighbors of *ctag1b/a*, *magec1* and *prame* in synovial sarcomas (*tle1*) as well as in ganglioneuromas (*ranbp2*), and confirmed the expression of the encoded proteins in independent samples. The network proximity of these genes in the *ctag1b/a* network, and by extension to the *ctag1b/a*, *prame* and *magec1* network clique (Figure 3B), suggests that although *ctag1b/a* expression is not uniform within the malignant tissue, it is likely involved in synovial sarcoma biology. Alternatively, re-expression could be the result of epigenetic factors. Rearrangement of their sub-networks reveals that the *ctag1b/a, magec1 and prame* clique (Figures 3B, 5) is maximally two degrees apart from *ranbp2, tle1, dyrk1a and six1* in the network, thus linking *cta1b/a* to the oncogenic pathways of *six1* (cyclinA1 or transcriptional misregulation in cancer via the Wnt/Hedgehog/Notch-mediated oncogenesis), dyrk1a (cell cycle control at the G1-G1/S phases), *tle1* (NFκB-mediated control of gene transcription), and *ranbp2* (mitotic cell cycle control and Notch pathways).

It is noteworthy that *ctag1b/a* is re-activated in CD133 positive but not in CD133 negative stem cells, suggesting that it could have an active role in cancer stem cell biology (Rojas-Benítez et al., 2013). Consistent with CTAG1B/A being a homolog of the yeast and Drosophila transcription factor PCC1P, the synovial *ctag1b/*a neighborhood is overwhelmingly enriched for transcription processes. Of the genes comprising the *ctag1b/a* network neighborhood, *ranbp2* and *six1* are directly linked in the *ctag1b/a*, *prame* and *magec1* clique (Figure 4B). Moreover, *ctag1b/a* is linked to a subnetwork of chromatin protein-encoding genes via *pasd1* which also encodes a putative transcription factor (Figure 3A). PASD1 is a cancer-associated antigen that can stimulate autologous T-cell responses, and it is therefore considered to be a potential immunotherapeutic target for the treatment of various hematopoietic malignancies (Scanlan et al., 2000; Scanlan et al., 2002a). Another gene of interest is *zic3* (Figure 3A) which encodes a member of the ZIC family of C2H2-type zinc finger proteins. ZIC3 probably functions as a transcription factor in early stages of left-right body axis formation. Mutations in this gene cause X-linked visceral heterotaxy, which includes congenital heart disease and left-right axis defects in organs.

The *ctag1b/*a sub-network neighborhood includes, among others, the *six1* gene (Figure 3A), a known oncogene that enhances tumorigenesis by activation of *cyclin a1* expression and *ranbp2*, a gene of the RAS super-family, that is, associated with the nuclear membrane and which immunolocalizes to the nuclear pore complex. It encodes a small GTP binding protein belonging to the RAS superfamily, that is, essential for the translocation of molecules through the nuclear pore complex. *Ranbp2* is implicated in different cellular functions through interactions with other proteins and has been implicated in the Ran-GTPase cycle. It directly interacts with the E2 enzyme UBC9 enhancing SUMO1 transfer from UBC9 to the SUMO1 target SP100. These findings place sumoylation at the cytoplasmic filaments of the nuclear pore complex suggesting that, for some substrates, modification and nuclear import are linked events. Notably, synovial sarcomas express the fusion oncoprotein SYT-SSX1 which enhances symoylation of NCOA3 through interaction with the SUMO E3 ligase, PIASy(Sun et al., 2011). Whether RANBP2 and SYT-SSX1 act in common sumoylation pathways remains to be found. Lastly, a RANBP2-ALK fusion oncoprotein is expressed in epitheliod inflammatory myofibroblastic sarcomas (eIMS) and combined targeting of CD30 and RANBP2-ALK shows therapeutic promise (Segal et al., 2004; Silva et al., 2007).

The vertebrate *six* genes are homologs of the Drosophila “sine oculis” (so) gene, which are expressed primarily in the developing visual system of the fly. Members of the *six* gene family encode proteins that are characterized by a divergent DNA-binding homeodomain and an upstream SIX domain, which may be involved both in determining DNA-binding specificity and in mediating protein-protein interactions. Genes of the *six* family are involved in vertebrate and insect development and in maintaining the differentiated state of tissues. Our results as well as those of others implicate Six1 in sarcoma growth (Stockert et al., 1998; Simpson et al., 2005; Sun et al., 2011) however its functional links to CTAG1B/A or to RANBP2 remain to be established and underscore the complexity of sarcoma biology.

*Tle1* is notable not only for being an expression neighbor of *ctag1b/a* in the GSEA-identified module but also a network neighbor that has been proposed as a diagnostic immunohistochemical marker for synovial sarcoma (Terry et al., 2007). Our network data suggest that ctag1a, magec1 and prame form a clique, where all three nodes are connected by edges to each other. Usually cliques are formed by functionally or physically and genetically linked genes. They could, therefore, affect each other’s biology in synovial sarcoma. PRAME is a known transcriptional repressor protein (Epping et al., 2005), that is, expressed in human melanomas and recognized by cytolytic T lymphocytes. Like CTAG1B/A (and MAGEC1), PRAME is not expressed in normal tissues, except in testis. The protein is a repressor of retinoic acid receptor, and confers a growth advantage to cancer cells *via* this function. CTAG1B/A and MAGEC1 proteins physically interact with each other in intracellular protein complexes and with a few RNA polymerase II subunits, thus raising the issue of whether they function as specialized transcription complexes in developing germ cells (spermatophytes and spermatids). Interestingly, expression of CTAG1B/A and PRAME has recently been correlated with tumor grade and poor prognosis in myxoid sarcomas, supporting our hypothesis that CTAG1B/A is active in sarcoma biology (Iura et al., 2015).

Our findings that the CTAG1B/A network is enriched with transcription factors (Figures 3C, 5) and its ability to compensate for the absence of the yeast transcription factor, PCC1P, support our hypothesis that CTAG1A complexes (likely with MAGEC1) are part of gametogenic germ cell transcription programs that could also be recruited to support tumor growth. Specifically, the PCC1P protein is part of an EKC/KEOPS multiprotein complex found in diverse organisms such as the Archaea and Drosophila (Hecker et al., 2008). Moreover, in yeast, this complex has been implicated in telomere maintenance, transcriptional regulation, bud site selection and chemical modification of tRNAs. In Drosophila, it has been linked to the TOR pathway via the protein kinase Bud32/PRPK, which regulates growth signals for nutrition status (Yu et al., 2018). Thus, the presence of *tle1* and *ranbp2* in the *ctag1b/a* network neighborhood tentatively link *ctag1b/a* to sarcoma biology.

## Conclusion

Using sarcoma transcriptomics data deposited in GEO, we have re-constructed a sarcoma *ctag1b/*a network, identified sub-network neighbors, and determined that it is enriched with transcription factor-encoding and chromatin-related genes. The combined data suggest that *ctag1b/*a is part of transcription complexes specific for germ cell development and differentiation. It is re-activated in advanced myxoid liposarcomas and synovial sarcomas but not in various differentiated liposarcomas or ganglioneuromas, thus indirectly implicating it in sarcoma biology. We have identified several genes in sarcoma samples that are network neighbors of *ctag1b/a* including *ranbp2*, *tle1, six1* and *prame* and using independent sarcoma samples, we immunohistochemically confirmed expression of the encoded proteins for *tle1* and *ranbp2*. Finally, the confirmation of expression of *ranbp2* and *tle1* as well as the presence of *six1* in its network neighborhood suggests that *ctag1b/a* may be part of transcriptional proteins that functionally interact with proteins involved in sumoylation (*via ranbp2*) and the cell cycle (*via six1, pasd1* and *zic3*) in sarcoma biology (Liu et al., 2018; Martínez-Arroyo et al., 2015; Ono et al., 2001; Türeci et al., 1998; Xu et al., 2015).

## Data Availability

The datasets presented in this study can be found in online repositories. The names of the repository/repositories and accession number(s) can be found in the article/Supplementary Material.

## Supplementary Material

Supplementary Figure S1
**(A)** Upper left panel. Top fifty genes identified with GSEA that show positive (CTAG1_POS) or negative (CTAG1_NEG) patterns of co-expression with ctag1b/a. Ctag1b/a (middle of Figure, left panel) was used as an index gene to compute the statistical distance of the other genes with GSEA as described in Computational section. Genes at the top and bottom are the most distant whereas those nearest ctag1b/a are those with the highest index of correlation (details in Supplementary Material). Upper right panels. Top enriched pathways of Ctag1_Positive or Negative modules computed as described in the Methods section. **(B)** Lower left panel: Heat map of gene expression status in phenotypic class lists of ctag1b/a co-expressed genes in microarray datasets retrieved from GEO (Supplementary File S1). Lower middle panel: Gene setsranked by Kolomogorov-Smirnov (KS) statistics resulting from comparison with the MSigDb (GSEA site, MIT) and with the Papanikolaou gene sets collection. Lower right panel: Computed ES score for genes lists and rank in ordered sets.
