# Supplementary material for: Systems-Level Mapping of Cancer Testis Antigen 1b/a to Sarcoma Pathways Identifies Activated Ran Binding-2 E3 SUMO-Protein Ligase and Transducin-Like Enhancer Protein 1
Source: Front Genet. 2022 May 18;13:834445. doi: 10.3389/fgene.2022.834445 (PMC9158115; doi:10.3389/fgene.2022.834445)
Supplement: Supplementary file 6 [file DataSheet1.PDF]

Suplementary pdf file

Appendix B contains the computational methods used to extract modules and to rank order gene sets and genes in the two classes of samples, i.e. *ctag1b/a*-expressing synovial and myxoid liposarcomas vs. benign liposarcomas.

Computational Methods

B1. Gene set enrichment analysis (GSEA)

In order to identify gene modules and top genes within them that might be co-regulated with CTAG1B/A in tumor samples shown in supplementary file 1 that express them versus those that do not, we employed the gene set enrichment analysis (GSEA) algorithm (Mootha et al., 2003;Subramanian et al., 2005) on genome-wide gene expression microarray data sets obtained from the Gene Expression Omnibus database (GEO) in two steps (See overall strategy in **Figure 1**): First, using Kolmogorov-Smirnov non-parametric statistics, all genes (features) in the datasets are ranked with respect to the two class vector samples that lack CTAG1B/A expression vs. samples that express CTAG1B/A, using CTAG1B/A as an index gene (i.e. its expression level of its probe set across all samples in the two classes, a continuous phenotype), and Euclidean distance as the gene distance metric (Expression datasets I with N genes and k samples). The same GSEA approach was applied to the datasets in order to retrieve the top 50 genes for the two classes of samples using as permutation metric the Phenotype differences (CT-X gene expression status classification) between the two (categorical phenotype analysis). In conclusion, gene modules are obtained (a) from samples where CTAG1B/A is either not expressed or expressed and used as an index gene to retrieve its co-regulated neighbors and (b) for the same sample classes gene modules that are enriched with respect to the index gene status.

For N genes in each class $c(k)$ *and* $t(k)$ represent the kth gene from the control and test populations respectively where $k=1,\ldots..K, \sum^{K/}{}_{k=1} c(k) = 1$, and $\sum t(k) = 1$. The cumulative distribution function (CDF) for the control and test classes are defined as $C(j)$ and $T(j)$ and computed with $C(j)= \sum k=_{1-J}\, c(k)$, $T(j) =_{1-J}\, t(k)$. Finally, the KS statistic, which is the maximal distance between the two CDFs is computed with $D = \max j \,|\, C(j)-T(j) \,|$ producing gene lists L (**Figure 2A**). The KS statistic is a quantitative measure of how likely the observed expression distribution differences between controls (tumor samples without expression, see **Figure 2A**) and those that express CTAG1B/A, is due to random chance.

B2. Enrichment analysis and ES computation

In the second step of gene set enrichment analysis (GSEA, **Figure 2A**), an algorithm which compares the position of genes in several hundred gene sets S (see www.broad.mit.edu/gsea/index.jsp for all gene sets used) within the ordered gene lists L derived from the microarray data, is executed and computes an enrichment score (ES) for each gene in S relative to its position in the ranked dataset list L and a normalized (NES) score (See Figure 3B for enrichment scores). Thus the algorithm computes whether genes in S are ranked high (positive ES) or low (negative ES) in L, and returns a numeric value (KS score, Supplementary file 3), which is different from the KS statistic discussed above and which represents gene ranking before enrichment) representing the positional distribution of the entire set of query genes in the two classes (**Figure 3A**). The KS score reflects the position of each gene within the ranked lists L and was computed in accordance to the non-parametric rank statistic X, where X is the number of genes in the query gene sets from control samples or samples with CTAG1B/A expression, Z is the number of genes in the ordered list (24000 features, see **Supplementary file 2** for detailed GSEA conditions and

a summary of the retrieved gene set numbers and gene set details), and Y=Z-X. The KS score for CTAG1B/A (or for any gene that serves as phenotypic index) is set to zero and the rank position in the ordered controls or samples that express CTAG1B/A of each gene is computed according to the formula given below. The ES score therefore is the maximum deviation from zero encountered in walking down the gene list L generated from the microarray datasets.

For categorical phenotype analysis (CTAG1B/A negative vs. CTAG1B/A positive+ samples) a positive ES score indicates correlation with the CTAG1B/A negative samples whereas a negative ES score indicates correlation with the CTAG1B/A positive samples. In contrast, in continuous phenotype analysis (CTAG1B/A as a gene index for co-regulated genes in either class of samples) a positive ES score indicates correlation with the CTAG1B/A_POS module and a negative one no correlation. The closest scores designate genes within the enriched gene sets that are closest in profile to the index gene (ESO) and the largest scores the most distant genes. The algorithm then constructs a vector V, where V(i) is the statistic corresponding to gene i in the ordered list having value V(i)=+Y, if gene i is in the gene set and V(i)=-X, if it is not. Also, false discovery rate (FDR) and p value statistics are computed for each gene in the ranked lists L for the two classes. Gene sets with significantly positive ES scores are those where V(i)=+Y whereas those with negative ES are V(i)=-X. Details on the gene collection sets used for this analysis can be found in the GSEA website (www.broad.mit.edu/gsea/index.jsp).

B3. Leading edge analysis

In order to retrieve the top genes that contribute to NES and that might affect more than one pathways regulated when CTAG1B/A is expressed, leading edge analysis of the gene sets with enriched GO annotation for co-regulation with CTAG1B/A in control or expressing samples was performed in GSEA.
